# Ca^2+^ influx-linked protein kinase C activity regulates the β-catenin localization, micromere induction signalling and the oral–aboral axis formation in early sea urchin embryos

**DOI:** 10.1017/S0967199414000033

**Published:** 2014-04-09

**Authors:** Ikuko Yazaki, Toko Tsurugaya, Luigia Santella, Jong Tai Chun, Gabriele Amore, Shinichiro Kusunoki, Akiko Asada, Tatsuru Togo, Koji Akasaka

**Affiliations:** 1 Department of Biological Sciences, Tokyo Metropolitan University, Minamiohsawa 1–1, Hachiohji-shi, Tokyo 192–0397, Japan.; 2 Department of Biological Sciences, Tokyo Metropolitan University, Minamiohsawa 1–1, Hachiohji-shi, Tokyo 192–0397, Japan.; 3 Misaki Marine Biological Station, University of Tokyo, Miura, Japan.; 4 Stazione Zoologica Anton Dohrn, Villa Comunale 1–80121 Napoli, Italy.; 5 LSL Co. Ltd, Nerima-ku Tokyo 178–0061, Japan.; 6 Department of Anatomy, St. Marianna University School of Medicine, Sugao, Kawasaki, Kanagawa 216–8511, Japan.

**Keywords:** β-catenin, Ca^2+^ influx, Oral–aboral axis formation, Protein kinase C, Sea urchin embryo

## Abstract

Sea urchin embryos initiate cell specifications at the 16-cell stage by forming the mesomeres, macromeres and micromeres according to the relative position of the cells in the animal–vegetal axis. The most vegetal cells, micromeres, autonomously differentiate into skeletons and induce the neighbouring macromere cells to become mesoendoderm in the β-catenin-dependent Wnt8 signalling pathway. Although the underlying molecular mechanism for this progression is largely unknown, we have previously reported that the initial events might be triggered by the Ca^2+^ influxes through the egg-originated L-type Ca^2+^ channels distributed asymmetrically along the animal–vegetal axis and through the stretch-dependent Ca^2+^channels expressed specifically in the micromere at the 4th cleavage. In this communication, we have examined whether one of the earliest Ca^2+^ targets, protein kinase C (PKC), plays a role in cell specification upstream of β-catenin. To this end, we surveyed the expression pattern of β-catenin in early embryos in the presence or absence of the specific peptide inhibitor of *Hemicentrotus pulcherrimus* PKC (HpPKC-I). Unlike previous knowledge, we have found that the initial nuclear entrance of β-catenin does not take place in the micromeres, but in the macromeres at the 16-cell stage. Using the HpPKC-I, we have demonstrated further that PKC not only determines cell-specific nucleation of β-catenin, but also regulates a variety of cell specification events in the early sea urchin embryos by modulating the cell adhesion structures, actin dynamics, intracellular Ca^2+^ signalling, and the expression of key transcription factors.

## Introduction

In sea urchin embryos, cell specifications take place along the two embryonic axes: the animal–vegetal (AV) axis and the oral–aboral (OA) axis. The AV axis can be traced back to the unfertilized egg (Boveri, 1901; Hörstadius, 1939), and the OA axis to the zygote (Coffman *et al*., 2004). By the end of the 4th division, three different sizes of blastomeres comprising eight mesomeres, four macromeres and four micromeres are formed and eventually become the ectoderm, the mesoendoderm and the mesoderm, respectively. The vegetal cells, micromeres, emit signals for the macromeres to form the endoderm (Ransick & Davidson, 1993), while they themselves develop into skeletogenic cells (Okazaki, 1975). It is generally accepted that these abilities of the micromeres coincide with nuclear localization of β-catenin at the 16-cell stage (Logan *et al*., 1999), although it is still unclear exactly how the β-catenin enters the nuclei (Davidson *et al*., 2002). β-Catenin is a multifunctional protein that is involved not only in cell–cell adhesion but also in gene expression (MacDonald *et al*., 2009). In epithelial cells, the cytoplasmic domains of cadherin link to the cortical actin cytoskeleton via α-, β- and γ-catenins (Takeichi, 1991; Gumbiner, 1996). In several cell types, the ‘myristoylated alanine-rich C kinase substrate’ (MARCKS) cross-links actin filaments and thereby anchors the actin network to the plasma membrane (Eliyahu *et al*., 2005). In sea urchin eggs, cadherin and β-catenin are co-localized in the cytoplasm and near the plasma membrane, and have been shown to accumulate at the sites of cell–cell contact following fertilization and cleavage (Miller & McClay, 1997a, b). Thus, the subcellular localization of β-catenin is a significant marker for the control of cell division and gene expression.

In many animals, inositol phospholipid turnover is involved in the regulation of many cellular processes. Phospholipase C (PLC) hydrolyses phosphatidylinositol 4,5-bisphosphate (PIP2) in the plasma membrane to produce 1,2-diacylglycerol (DAG) and inositol 1,4,5-trisphosphate (InsP_3_). It has been shown that DAG activates protein kinase C (PKC), and that InsP_3_ promotes an increase in intracellular Ca^2+^ concentration (Nishizuka, 1984; Berridge, 1993). PKC from sea urchin embryos was cloned from *Lytechinus pictus* (Rakow & Shen, 1994), and its roles have been well demonstrated during egg activation (Shen & Buck, 1990), skeletogenesis (Mitsunaga *et al*., 1990) and apoptosis in gastrulae (Dickey-Sims *et al*., 2005). Livingston & Wilt (1992, 1995) suggested that the InsP_3_-PKC signalling pathway played an important role in vegetalizing the ectodermal cells by showing that 10–15 min exposure of the embryos at the 16-cell stage to a PKC activator 12-*O*-tetradecanoyl phorbol-13-acetate (TPA) is sufficient to vegetalize the embryos. In addition, the presumptive ectoderm blastomeres could be vegetalized by a lithium ion (Li^+^) that modulates the metabolism of inositol phospholipids, and the vegetalizing effect of lithium was reversed by preinjection of the blastomere with myo-inositol (Livingston & Wilt, 1995). However, Li^+^ is also known to inhibit glycogen synthase kinase 3 (GSK-3), which regulates the activity of the transcription factors and β-catenin (Li *et al*., 2007), and to increase the levels of DAG and PKC activity (Drummond and Raeburn, 1984).

Sea urchin embryos exhibit polarized Ca^2+^ influxes in every cell division after fertilization (Yazaki *et al*., 1995; Dale *et al*., 1997). The micromere-specific Ca^2+^ influxes occur at the 4th cleavage and are followed by Ca^2+^oscillation for at least 10 min (Yazaki, 2001; Yazaki *et al*., 2004). It is plausible that these Ca^2+^ influxes or their amplified signals could lead to PKC activation. In the present paper, to elucidate the role of PKC activity in the functions of micromere and in the development of embryo, we have used a PKC pseudosubstrate inhibitor (HpPKC-I) constructed from the amino acid sequence of the *Hemicentrotus pulcherrimus* PKC and a specific antibody against β-catenin of *H. pulcherrimus* (Hpβ-catenin). First of all, contrary to a previous report (Logan *et al*., 1999), we found that the initial nuclear entrance of β-catenin takes place in the macromeres, not in the micromeres, as judged by the co-staining of β-catenin and the DNA-binding dye propidium iodide (PI). Secondly, we have found that the inhibition of PKC leads to appreciable deregulation of the cortical actin cytoskeleton in the blastomeres. In addition, we have examined the morphology and the gene expression pattern of embryos after the treatment of with HpPKC-I in the early cleavage stage. We have found that: (i) PKC activity regulates the nuclear localization of β-catenin by means of the cortical actin filaments; (ii) PKC controls the early endoderm induction signal of micromeres by modulating the intercellular adhesion system and intracellular Ca^2+^ signalling; and (iii) the nuclear β-catenin activates not only the endodermal genes but also ectodermal genes, depending on their timing and location, without interfering with the formation of the animal–vegetal and oral–aboral axes.

## Materials and methods

### Animals and gametes

Individual animals of *Hemicentrotus pulcherrimus* were supplied from the Misaki Marine Biological Station, the University of Tokyo, and *Paracentrotus lividus* from the Stazione Zoologica Anton Dohrn in Naples, Italy. Gametes were obtained by intracoelomic injection of 0.5 M KCl for *H*. *pulcherrimus*. *P. lividus* eggs were released from the dissected gonad into in natural seawater filtered with a Millipore-filter set. Eggs were washed and inseminated in seawater that contained 5 mM p-aminobenzoic acid (PABA-SW). Fertilization envelopes were removed by pipetting, and the embryos were cultured in filtered seawater at 15°C or 18°C (*H*. *pulcherrimus*), or at room temperature (20–23°C) for *P. lividus*.

### Preparation of HpPKC inhibitor (HpPKC-I) and anti-Hpβ-catenin antibody

The HpPKC pseudosubstrate domain (FARRGALRQ) was synthesized according to GenBank information on *H. pulcherrimus PKC* (AB699356, amino acids 11–19) and myristoylated at the N-terminus of the peptide (Peptide Institute Inc., Osaka, Japan) to enable it to permeate cell membranes (Eichholtz *et al*., 1993). Recombinant protein of Hpβ-catenin was expressed bacterially by using the sequence data from the cDNA clone (AB699355) isolated from the *H. pulcherrimus* gastrula cDNA library (Fuchikami *et al*., 2002). The cDNA fragment encoding the N-terminal region of Hpβ-catenin (1–250 amino acids) was inserted into the pET42b/GST vector and expressed in *E. coli* BL21 (Stratagene). The overexpressed protein was extracted and purified by affinity chromatography using a ‘GST-Bind Resin’ column (Novagen). The GST-tag was removed using the Thrombin Cleavage Capture Kit (Novagen), and the remaining polypeptide was used to immunize rabbits to generate polyclonal antibodies against Hpβ-catenin.

### Immunoblotting

Whole lysate of *H. pulcherrimus* embryos was prepared by sonication in Laemmli's sample buffer (62.5 mM Tris–HCl, pH 6.8, 2% sodium dodecyl sulphate (SDS), 10% glycerol, 0.005% bromophenol blue, 5% β-mercaptoethanol) and boiled for 5 min. SDS-PAGE was carried out with 10% polyacrylamide gels. Proteins were transferred to polyvinylidene difluoride (PVDF) membranes (Millipore, Bedford, MA, USA) using a semi-dry blotting apparatus. Membranes were probed with the rabbit antiserum against Hpβ-catenin (1:10,000 dilution) and the horseradish peroxidase (HRP)-conjugated swine anti-rabbit IgG (Dako, Glostrup, Denmark). Immunoreactivity signals were detected with the Millipore Immobilon western chemiluminescent HRP substrate (Millipore).

### Whole-mount immunostaining

Following the methodology by Logan *et al*. (1999), embryos of *H. pulcherrimus* were fixed in artificial seawater (ASW; 425 mM NaCl, 9.3 mM KCl, 10 mM CaCl_2_, 24.5 mM MgCl_2_, 25.5 mM MgSO_4_, 2.5 mM NaHCO_3_, pH 8.0) containing 2% paraformaldehyde for 10 min at room temperature, and then passed briefly through 100% MeOH on ice to permeabilize the cell membrane. After three cycles of washing in phosphate-buffered saline (PBS) by sedimentation, embryos were treated with 3% bovine serum albumin in PBS containing 0.1% Tween 20 (TPBS) for 30 min, and then incubated with rabbit polyclonal antiserum against Hpβ-catenin (1:10,000 dilution) for 1 h with gentle agitation. Following one wash in TPBS and three subsequent washes in PBS, embryos were incubated for 1 h in a solution that contained the secondary antibody: the Alexa Fluor 488-conjugated anti-rabbit IgG (Molecular Probe) at 1 μg/ml (1:2000 dilution of the stock solution) in 0.1 M Na-phosphate, 0.1 M NaCl, 5 mM NaN_3_, pH 7.5. In the last10 min of the immuno-reaction, a DNA-binding dye PI (KPL) was added at the 1:600 dilution ratio of the stock solution (1 mg/ml PBS). After four washes in PBS, the specimens were placed in a well on a glass slide surrounded by lens paper soaked in mineral oil and were monitored with an Olympus laser-scanning confocal microscope (LSM-GB200).

### Visualization of F-actin

*P. lividus* embryos were used for the observation of F-actin in accordance with the method described previously (Kyozuka *et al*., 2008). Control embryos and the embryos treated with PKC modulators were fixed for 30 min in seawater that contained 4% formaldehyde and 0.1% glutaraldehyde, starting from 20 min after the 4th cleavage. After three cycles of rinse in the washing buffer (50 mM HEPES, pH 7.0, 50 mM PIPES, 600 mM mannitol, 3 mM MgCl_2_) that contained 0.1% Triton X-100, the embryos were incubated for 30 min with 3–10 U/ml of Alexa Fluor 488-conjugated phalloidin in PBT (137 mM NaCl, 2.7 mM KCl, 15 mM KH_2_PO_4_, 8 mM NaHPO_4_, 0.1% Triton X-100, pH 7.2) and then rinsed in PBT for three times. All steps were performed at room temperature. The embryos stained with fluorescent phalloidin were observed under an Olympus Fluoview 200 laser-scanning microscope using the BP510540 emission filter. Transmitted light and fluorescent images were acquired from the same confocal plane.

### Chemical treatments of embryos

LiCl was added to the 4-cell stage *H. pulcherrimus* embryos at the final concentration of 40 mM as described by Ciapa & Maggio (1993), and a batch of the embryos were grown up to the 16-cell stage (2.5 h) or until vegetalization (5 h) in the LiCl-seawater. PKC activator, phorbol-12-myristate-13-acetate ester (PMA; CALBIOCHEM) was added 5 min before the 4th cleavage at the final concentration of 8 nM (Livingston & Wilt, 1992) and incubated for 20 min. HpPKC-I was added to the embryos 20 min before the 4th division until 20 min after the 6th division with the final concentration of 5–7 μM. A Ca^2+^-dependent PKC-specific inhibitor, 12-(2-cyanoethyl)-6,7,12,13-tetrahydro-13-methyl-5-oxo-5-idolo (2,3-a)pyrrolo(3,4-c)-carbazole (Gö6976; CALBIOCHEM) was applied at a concentration of 400 nM during the same period as the treatment with HpPKC-I. Gadolinium chloride (GdCl_3_·6H_2_O; Sigma), a stretch-activated calcium channel blocker (Yang & Sacks, 1989), was used for suppression of Ca^2+^ elevation in the micromeres (Yazaki *et al*., 2004) by application at the concentration of 10–30 μM from 10 min before the 1st, 3rd, 4th and 7th cleavages, respectively.

### Ca^2+^ measurements

*Paracentrotus lividus* eggs were dejellied with acid seawater and washed in 10 ml seawater that contained 3 mg *p*-aminobenzoic acid (PABA; Sigma) and then lined on a protamine sulphate-coated dish filled with PABA seawater. A mixture of 10 mg/ml of calcium green® BAPTA conjugated with 10 kDa of dextran (Molecular Probe) and 3 mg/ml of rhodamine red was prepared in the injection buffer (0.45 M KCl, 10 mM HEPES, pH 7.0), and microinjected into eggs within 5 min after fertilization. The eggs were then collected by a mouth pipette and transferred to agar-coated dishes filled with Millipore-filtered natural seawater. About10 min before the 2nd cleavage, zygotes were transferred into the Ca^2+^-free seawater (CFSW; 530 mM NaCl, 10 mM KCl, 27 mM MgCl_2_, 29 mM MgSO_4_, 2 mM NaHCO_3_, pH 8.0). Blastomeres were separated at the 4-cell stage by gentle pipetting. These cells give rise to quarter embryos, which are able to configure their cells as 1-cell layer: two mesomeres, one macromere and one micromere to enable photometry on each cell. Ca^2+^ imaging was performed in the period from 5 to20 min after the 4th cleavage, the time of the micromere-specific Ca^2+^elevation had established in normal embryos, using a charged coupled device (CCD) camera with a ×63 objective and MetaMorph analyzing software. Calcium levels were expressed as the ratios of fluorescence intensities between calcium green® BAPTA (Ex 485 nm/Em 535 nm for 200 ms) and the internal control rhodamine red (Ex 555 nm/Em 565 nm for 100 ms exposure). When a time course measurement was needed, the time resolution was 5 s.

### RT-qPCR analysis

Total RNA was extracted from *Strongylocentrotus purpuratus* embryos at pertinent developmental stages with the RNeasy mini kit columns (Qiagen), and subjected to cDNA synthesis by use of the SuperScript First-Strand Synthesis System (Invitrogen). Polymerase chain reaction (PCR) was carried out with specific primers synthesised from mRNA sequences of target genes in *Strongylocentrotus purpuratus*. The sequences (5′→3′) of each primer set (forward and reverse) are as follows: *Gsc*, F_ggattgatggcgatacgact and R_gcagaggaagacaccgagac; *Delta*, F_tgtgcaataaccagggtgaa and R_tgcaagagtttggtgagtcg; *Nodal*, F_gacctgctctggacctcttg and R_agtggtcgaagtttggttgg; *Lim*, F_gtatctttcgggcaaggaca and R_ctcttccatcggatgtggat; *Gcm*, F_atcgttcaccatcgacaaca and R_aggcatggtaggcagctaag; *FoxG*, F_gtgaaagtccctcgccatta and R_tcagcttacccgttgtaccc; *Brain 1/2/4*, F_gacgcattaagctcggctac and R_aagctcaactgcaaggcttc; *FoxA*, F_caattcgccacagtctctca and R_ctcggacatattcccagcat; NK22, F_actttggttcggttggtcag and R_ttcacggaaaagctcgtctt; *Coquilette*, F_ctgaccaggcatttcatgtg and R_gtgcaccagggcactatttt; *Blimp*, F_cagggaatctccgattcaaa and R_tggcacctcctcttctctgt; *Deadringer*, F_cagagctgcaatcagccata and R_gggaaccagaaggggtatgt; *Sm 30*, F_caaccagtctggatcggtct and R_ataggggccatcattccttc; *Sm 50*, F_gccttttcgcaggataatca and R_ccgtttggataagctggtgt; *G*-*cadhedrin*, F_cggtggaacttgtgttgatg and R_cgtaaccgttgttgaagctg; *Bra*, F_aggacacggaattgtggaag and R_gcgatgttagccgagagaac. The automated thermal cycler used was the Chromo4™ Real-Time Detector (Biorad) utilizing Sybr Green in the reaction mixture that is intercalated into double-stranded PCR amplicons and emits fluorescence. For each query transcript, the cycle threshold value (C_t_) was normalized to the C_t_ value obtained on the same cDNA preparations with ubiquitin primers: i.e., C_t_ = C_t (sample)_/C_t (ubiquitin)_. To evaluate the effect of the PKC modulator on the transcript level of each query gene, the difference (D) between the C_t_ values of the control and experimental samples were obtained, i.e., D = C_t (control)_ – C_t (experimental)_. Thus, positive D values indicate an increase in the transcript after treatment. To convert D into the ratio (R) of the transcript concentrations, we applied the formula R = 1.94 × D, where 1.94 is the mass amplification coefficient per PCR cycle for this particular case.

### Statistical analysis

Paired *t*-test and one-way analysis of variance (ANOVA) with Tukey's multiple comparison test were performed using Prism 6.0 software (Graph Pad Software); *P*-values less than 0.05 were considered to be statistically significant.

## Results

### Specificity of the sea urchin-specific PKC inhibitor and anti β-catenin antibody

**Figure 1:**
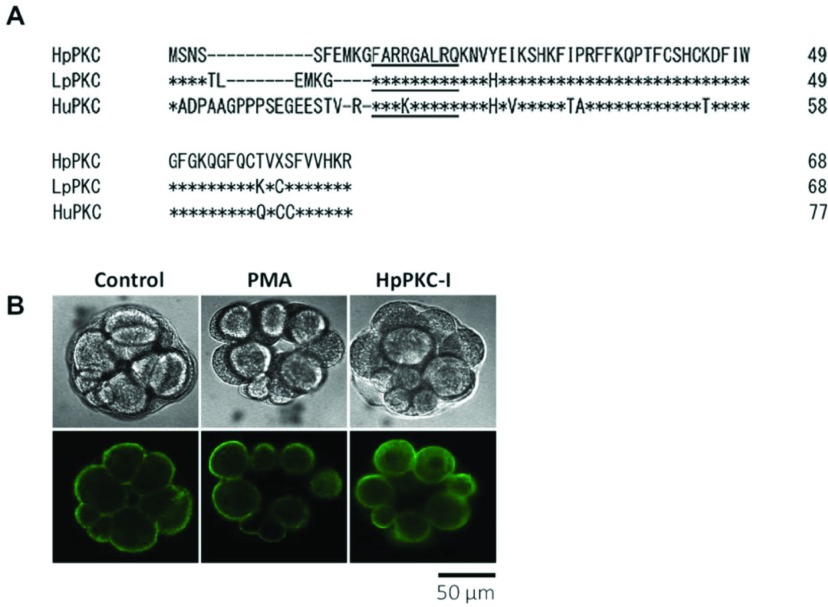
Inhibition of PKC interferes with the normal distribution of actin cytoskeleton in blastomeres. (*A*) Preparation of the HpPKC inhibitor (HpPKC-I). The amino acid sequence of *Hemicentrotus pulcherrimus* PKC was aligned with *Lytechinus pictus* (LpPKC) and human PKC (HuPKC) (1–68) to define the pseudosubstrate region (underlined nine residues). Asterisks denote identical amino acid residues. The synthetic peptides of the pseudosubstrate were myristoylated to use as HpPKC-I. (*B*) Effects of PKC on actin distribution. F-actin was visualized with Alexa Fluor 488-conjugated phalloidin; 8 nM PMA was added to the embryos (*P. lividus*) for 20 min starting from 7 min before the 4th division; 5 μM of HpPKC-I was added 20 min before the 4th division. Optical (upper) and fluorescent images (lower) were taken from the same confocal plane. In either case, cell specifications were modified, but cell division progressed normally.

**Figure 2:**
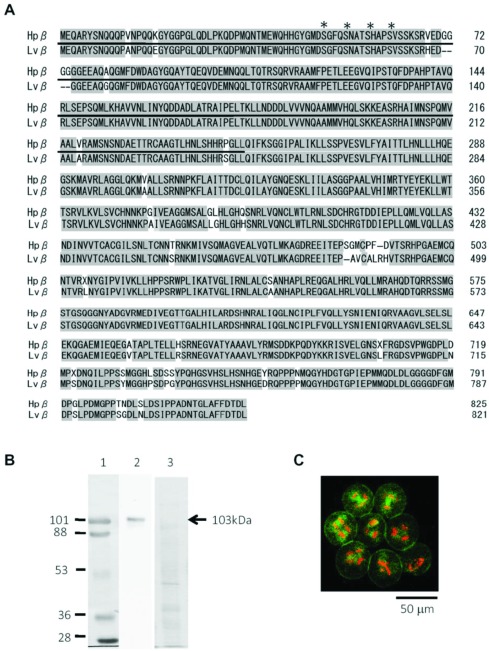
Specificity of the antibody against Hpβ-catenin. (*A*) Deduced amino acid sequences of β-catenin from *H. pulcherrimus* (Hpβ) and *Lytechinus variegatus* (Lvβ; Miller & McClay, 1997b) were aligned. Hpβ-catenin and Lvβ-catenin were 95% identical (shaded amino acid residues). The consensus serine/threonine targets for GSK-3β were marked with asterisks. Hpβ-catenin antibody was prepared by using the underlined region (1–250 amino acids), which included the epitope for Lvβ-catenin antibody (1–173 amino acids). (*B*) Western blot analysis with anti-Hpβ-catenin antibody: lane 1, molecular weight marker; lane 2, cell lysate prepared from the fertilized eggs of *H. pulcherrimus*; lane 3, the blot stained with Ponceau-S. Hpβ-catenin protein sized 103 kDa was marked with an arrow. (*C*) 7 μM HpPKC-I inhibits cell division. HpPKC-I was applied to *H. pulcherrimus* embryos from 20 min before the 4th cleavage. Embryos were fixed when untreated embryos arrived at the 32-cell stage, and stained with the DNA dye PI (red) and Hpβ-catenin antibody (green). Cytokinesis was blocked, but nuclear division progressed normally.

To study the roles of the PKC pathway in the early development of *Hemicentrotus pulcherrimus* embryos, we prepared a specific peptide inhibitor of PKC and antibody against β-catenin, which is a potential downstream target of PKC in the micromeres (Livingston & Wilt, 1992, 1995; Wikramanayake *et al*., 2004). As described in Materials and methods, the peptide inhibitor elected from *H. pulcherrimus* PKC (HpPKC) was completely identical to *Lytechinus pictus* PKC (LpPKC; Rakow & Shen, 1994), differing from the human PKC pseudosubstrate by only one amino acid (Fig. 1*A*). When HpPKC-I (7 μM) was applied to the embryo 20 min before cleavage, the next cell division stopped, although the nuclear division progressed at the normal schedule (Fig. 2*C*). At a lower dose (5 μM), we found that treatment with HpPKC-I strikingly reorganized the actin cytoskeleton in the blastomeres. Whereas the Alexa 488-phalloidin-stained F-actin filaments in the normal blastomeres were localized predominantly at the plasma membrane, the distribution of F-actin in the blastomeres of the HpPKC-I-treated embryos was shifted more towards the cytoplasm (Fig. 1*B*). In contrast, when the myristoylated pseudosubstrate of human PKC differing by only one amino acid was used (Calbiochem), cell division of sea urchin embryos was not affected, even at 28 μM (data not shown). This finding suggested that the inhibitory effect on the target enzyme requires highly stringent specificity.

As to the specificity of the anti-Hpβ-catenin antibody, we used a part of the Hpβ-catenin as the epitope. To this end, the homologous protein to *Lytechinus variegatus* β-catenin (Lvβ-catenin) (Miller & McClay, 1997a) was cloned and verified by sequence comparison. As shown in Fig. 2*A*, the amino acid sequence of Hpβ-catenin (825 amino acids) was 95% homologous to that of Lvβ-catenin (821 amino acids). Antibody to Hpβ-catenin was prepared against amino acids 1–250, in consideration of the antigenic region of Lvβ-catenin (1–173 amino acids, underlined). In this region, four residues of serine/threonine (asterisked) matched the consensus substrate sequences of the glycogen synthase kinase-3β (GSK-3β) (Fig. 2*A*). As expected, when a western blot of the proteins extracted from *H. pulcherrimus* eggs was probed with the Hpβ-catenin antibody, a single band of 103 kDa was recognized as Hpβ-catenin (Fig. 2*B*). To the best of our knowledge, this finding is the first information for the molecular weight of sea urchin β-catenin. Taken together, these results assured that our PKC inhibitor and anti β-catenin antibody were highly specific.

### Hpβ-catenin initially nucleated in the macromeres at the 16-cell stage

**Figure 3:**
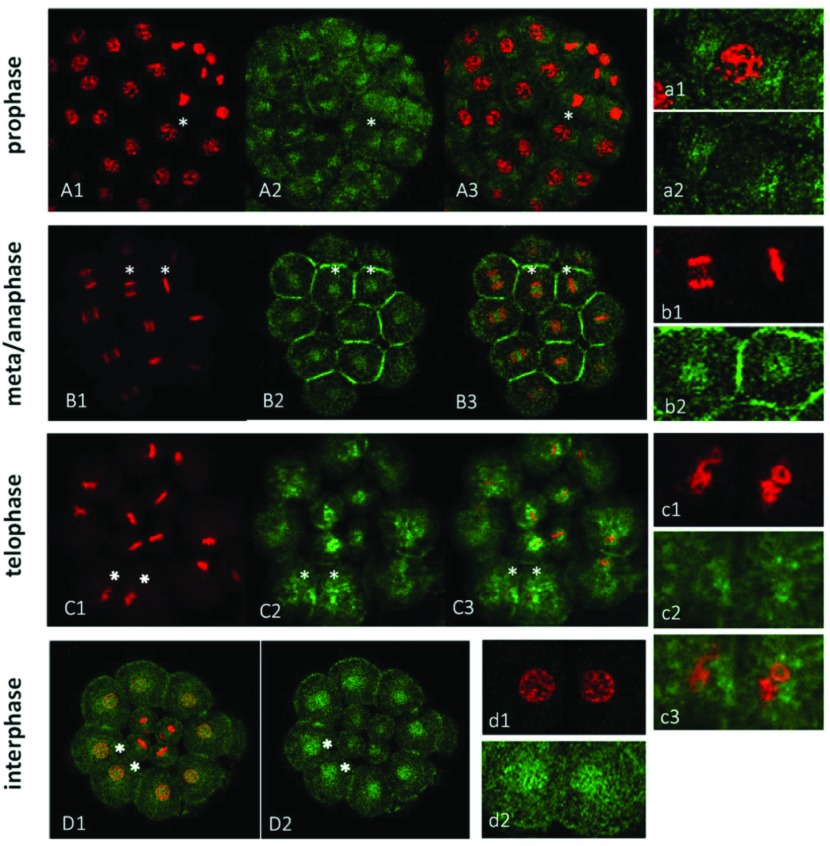
Distribution of Hpβ-*catenin* during mitosis. Embryonic cells at various mitotic stages (*H. pulcherrimus*) were stained with Hpβ-catenin antibody (green) and PI (red). Cells marked with asterisks in each embryo of (*A*), (*B*), (*C*) and (*D*) respectively were enlarged in the right-side panel (*a*, *b*, *c*, *d*). (*A*) At the 56-cell stage, most macromere and all mesomere derivatives shown were in prophase. (*B*) Cells at metaphase and anaphase in the 28-cell stage embryo. (*C*) Cells at telophase in the 28-cell stage embryo from the ventral view. Macromeres have just divided to be in telophase, showing an irregular, chambered structure in the process of reconstruction of the daughter nuclei. (*D*) A ventral view of 28-cell stage embryo. Macromeres were at interphase.

In the normal sea urchin embryos, we found Hpβ-catenin protein localized in the cytoplasm and in the apical and intercellular regions of plasma membrane by the 8-cell stage (Fig. 2*C*). This result is consistent with immunostaining with anti-Lvβ-catenin antibody (Miller & McClay, 1997a). From the 16-cell stage, the cell cycle of the embryo became asynchronous; the micromeres took longer time to arrive at the next (5th) cleavage than other blastomeres. To monitor the mitotic phase of blastomeres, every embryo stained with Hpβ-catenin antibody was counterstained with a DNA-binding dye PI. At prophase, the PI stain displayed a thread or spireme to construct chromosomes (Fig. 3*A1*, *a1*). Interestingly, Hpβ-catenin appeared to be concentrated at the place where amphiaster is located (Wilson, 1937). At metaphase, the chromosomal plate was centred while spindles were formed. At anaphase, chromatids were separated within the spindles (Fig. 3*B1*, *b1*). We found Hpβ-catenin either surrounding the equatorial plate of metaphase chromosomes (the right cell marked with asterisk in Fig. 3*B1*, *B2*) or localized in the space between the separating chromosomes (the left cell marked with asterisk in Fig. 3*B1*, *B2*). In the latter case, β-catenin was scarce near the mitotic asters. In Fig. 3*C*, four micromeres in the centre were evident with metaphase chromosomes, but the eight surrounding cells were already in telophase. When the chromosomes construct the nuclei in the newly divided cells at telophase, the Hpβ-catenin was evidently outside the constructing nuclei (Fig. 3*c1*–*c3*). In Fig. 3*D*, the outer eight macromere derivatives are in interphase, and Hpβ-catenin accumulated in the interphase nuclei (Fig. 3*d1*, *d2*). Hpβ-catenin changed its subcellular distribution during mitosis presumably by its interaction with the actin cytoskeletons through cadherin (Bienz, 2005) rather than with microtubules. Indeed, Hpβ-catenin immunoreactivity was present in the spindle regions but absent in the astral regions (Fig. 3*B*, *b*).

**Figure 4:**
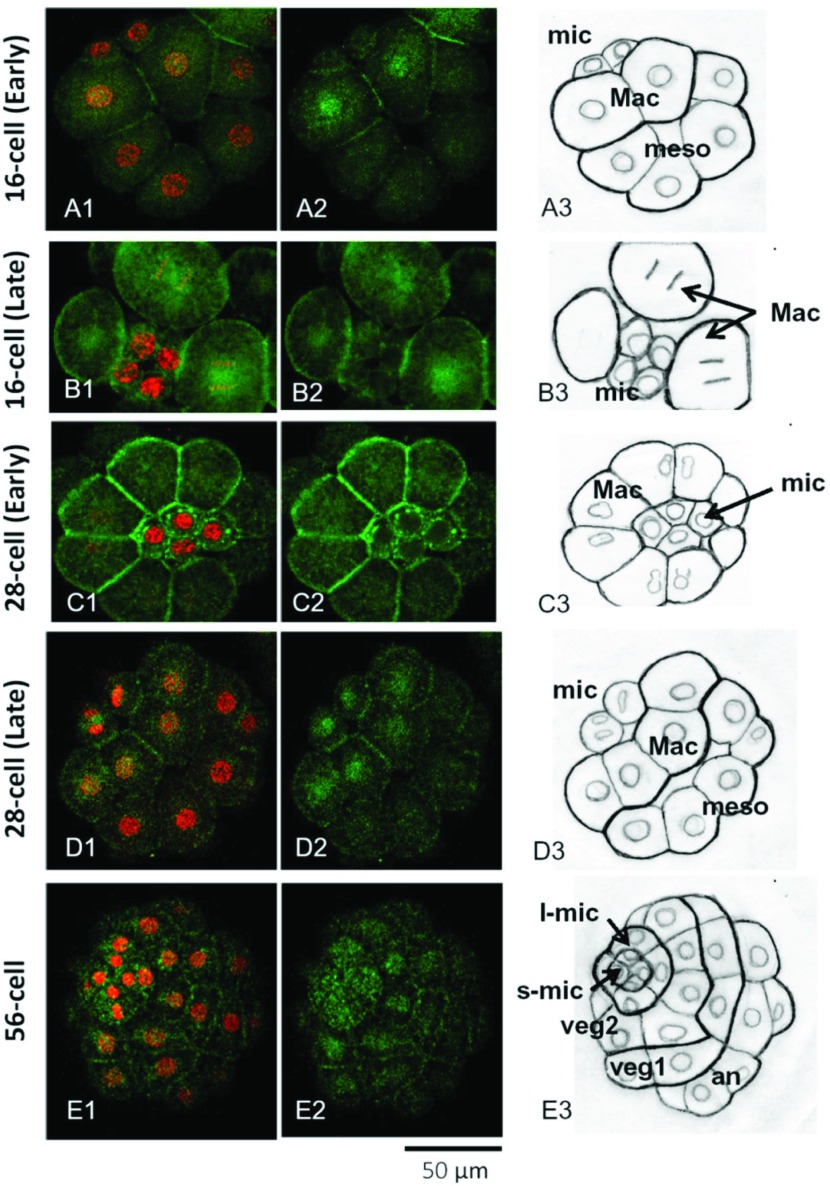
Localization of the nuclear β-catenin in the 16-cell to 56-cell stage embryos. (*A1–E1*) double staining of propidium iodide (PI) (red) and β-catenin antibody (green). (*A2–E2*) images of β-catenin staining only. (*A3–E3*) Drawing of (*A1–E1*) images to illustrate the nuclei and the contour lines of blastomeres. Abbreviations: mic; micromeres, Mac; macromeres, meso; mesomeres. (*A*) *H. pulcherrimus* embryo at the 16-cell stage: all cells were at interphase. Nuclear β-catenin was preferentially present in Mac. (*B*) Late 16-cell stage: mic remained at the interphase, but Mac progressed to anaphase. No β-catenin was found in mic nuclei. (*C*) Early 28-cell stage: Mac divided to the telophase, and mic formed chromosomal plate of metaphase. (*D*) At the 28-cell stage: mic at metaphase or anaphase. Mac and meso derivatives were at interphase. Nuclear β-catenin was detected only in Mac derivatives. (*E*) At the 56-cell stage: s-mic, small micromeres; l-mic, large micromeres. Veg1 and Veg2 are the macromere derivatives locating at the animal side (an) or next to l-mic, respectively. All cells shown were at interphase, and nuclear β-catenin was detected in every cell except for ‘an (animal side)’ cells. Abbreviations: An, animal side; Vg, vegetal side.

Figure 4 shows the Hpβ-catenin distribution in embryos from the 16- to 56-cell stages. At the 16-cell stage, nuclear β-catenin was usually detected only in the macromeres (Mac) (Fig. 4*A*), although in some batches of embryos it was detectable also in the micromeres, but its fluorescence intensity was always much weaker than in the macromeres (data not shown). In the late 16-cell stage (Fig. 4*B*), the macromeres and mesomeres entered the next cleavage cycle (anaphase chromosomes were visible in Mac) and the four micromeres (mic) were still in the interphase. At the early 28-cell stage, the micromeres were in metaphase with more condensed stain with PI, while Mac had already divided and attained the telophase. It is noticeable that β-catenin accumulated around the newly formed metaphase chromosomal plates of mic (Fig. 4*C1*, *C2*). When the Mac and meso had completed the 5th division and formed interphase nuclei, the micromeres belatedly entered anaphase of the 5th division, displaying nuclear-β-catenin-like distribution (Fig. 4*D*). Nuclear β-catenin was still detected only in the daughter cells of the macromeres. At the 56-cell stage (Fig. 4*E*), four small (s-mic) and four large micromeres (l-mic) were formed, and the macromere descendants were located in the veg1 and veg2 cell layers. Nuclear β-catenin was now detected in the interphase nuclei of both s-mic and l-mic, and as well as in the veg2 cell layer. A small weak signal seemed to be present in the nuclei of veg1 cells. However, nuclei of animal cells (an) always exhibited no β-catenin signal.

### PKC regulates nuclear entrance of Hpβ-catenin

**Figure 5:**
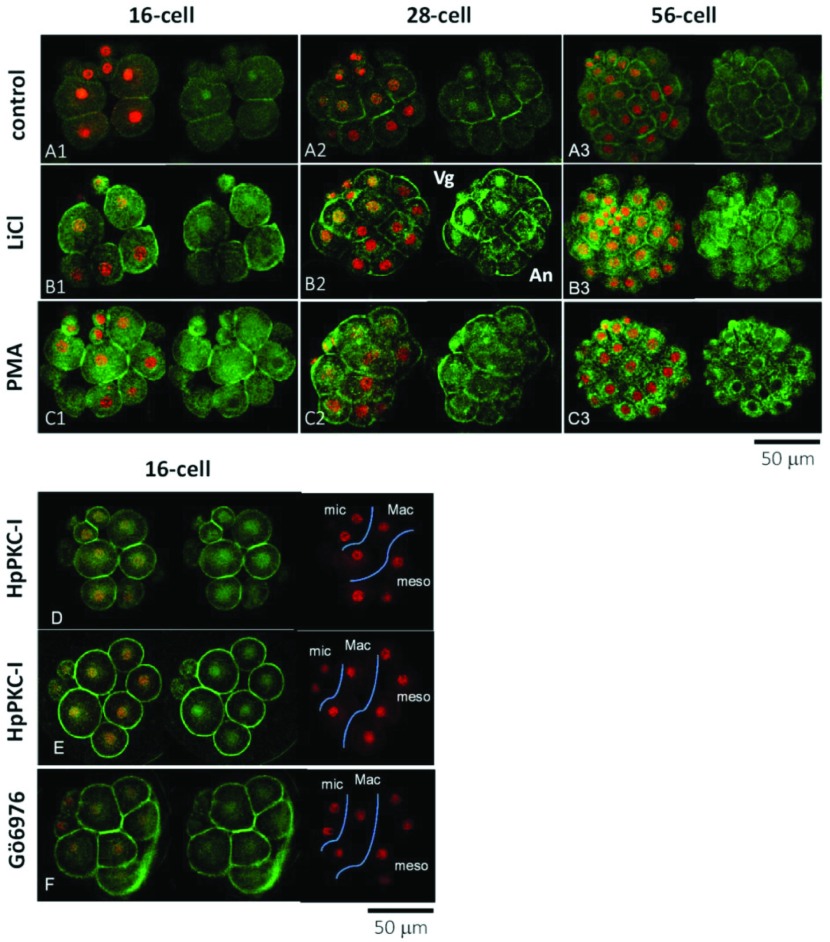
LiCl, PKC activator and PKC inhibitors affect the distribution of β-catenin. Localization of β-catenin (green) and nuclear staining (red) in the *H. pulcherrimus* embryos at the 16-cell, 28-cell and 56-cell stages. (*A*) Control embryos. (*B*) Embryos treated with 40 mM LiCl starting from the 4-cell stage. (*C*) Embryos exposed to PKC activator (8 nM PMA for 20 min starting from 5 min before the 4th cleavage). (*D*, *E*) Embryos exposed to 5 μM HpPKC-I starting from 20 min before the 4th division (16-cell stage). (*F*) Embryos exposed to 400 nM Gö6976, a calcium-dependent PKC inhibitor, for the same period as HpPKC-I. Abbreviations: An, animal side; Vg, vegetal side; mic; micromeres, Mac; macromeres, meso; mesomeres.

In the 28-cell embryo, nuclear localization of the β-catenin was evident preferentially in the cells at the vegetal side, although the micromeres had not yet arrived at interphase and therefore exhibited no clear sign of nuclear β-catenin (Fig. 4). When the embryos were treated with LiCl, which is a well known vegetalizing agent, the overall immunoreactivity of β-catenin was increased throughout the cytoplasm (Fig. 5*B*), and the nuclei were more densely stained in the cells at the vegetal side in comparison with the control, although the nuclei of the cells in the animal side were largely devoid of β-catenin (Fig. 5*B2*; Logan *et al*., 1999). Similarly, addition of the activator of PKC (phorbol 12-myristate 13-acetate, PMA; also known as TPA) markedly increased β-catenin immunoreactivity in the cytoplasm and nucleus, and the cells in the animal side were largely missing β-catenin immunoreactivity in the nucleus (Fig. 5*C*). Thus, the addition of LiCl and PMA appeared to intensify the AV gradient of nuclear β-catenin, which is found in the control. By contrast, 5 μM HpPKC-I markedly increased β-catenin predominantly near the plasma membrane with the concomitant decrease in the cytoplasm. In addition, it was noted that β-catenin was present in the nuclei of all blastomeres (Fig. 5*D*, *E*). Embryos treated with another PKC inhibitor Gö6976 produced virtually the same results as in the embryos treated with HpPKC-I (Fig. 5*F*). Hence, whereas a pharmacological activator of PKC mimics some of the effects displayed by Li^+^ in terms of β-catenin distribution, the peptide inhibitor of the PKC interfered with the normal subcellular distribution of β-catenin, negating its cell-specific nuclear localization.

### Effects of HpPKC inhibitor on micromere-specific Ca^2+^ rise and gastrulation

Sea urchin embryos display two types of Ca^2+^influxes. L-type Ca^2+^ channels derived from the egg plasma membrane function from the first cleavage and show AV axis polarity (Yazaki *et al*., 1995). Ca^2+^ currents were highest in mesomeres, but nearly absent in micromeres. Another type of Ca^2+^ influx starts to take place at the 4th cleavage through a stretch-dependent Ca^2+^ channels in the micromere (Yazaki, 2001). This micromere-specific Ca^2+^influx was followed by Ca^2+^oscillations through the InsP_3_/PKC signalling pathway, and thereby attained the intracellular Ca^2+^ level in the micromeres to about 10 % higher than in other blastomeres (Yazaki *et al*., 2004). While the Ca^2+^ increase is expected to activate the PKC pathway, it is not well known whether PKC in turn may contribute to modulate the intracellular Ca^2+^ levels in the micromeres. Here we have tested if PKC plays a role in the micromere-specific Ca^2+^ elevation. Using a fluorescent Ca^2+^ indicator (see Materials and methods), we have monitored changes in the intracellular Ca^2+^ levels 5–10 min after the 4th cleavage. The Ca^2+^ levels of each blastomere were monitored by single photometry at the 16-cell stage (Table 1). As expected from a previous study using intracellular recording (Yazaki, 2001), the Ca^2+^ levels in the micromeres (0.99 relative fluorescence unit; RFU) were slightly, but significantly, higher than those of the macromeres (0.91 RFU, p < 0.05) and the mesomeres (0.92 RFU, p < 0.05). In embryos treated with the PKC activator PMA (8 nM), the intracellular Ca^2+^ levels in all blastomeres were significantly lowered in comparison with the control (*P* < 0.05; Table 1). In line with this result, the addition of 400 nM Gö6976 (a pharmacological inhibitor of PKC) significantly increased the Ca^2+^ levels in all blastomeres, and suggested that PKC is involved in intracellular Ca^2+^ homeostasis. Interestingly, when the embryos were treated with 5 μM HpPKC-I, the disparity of the Ca^2+^ levels in the three types of blastomeres was abolished, as the level in the micromere was reduced significantly (*P* < 0.05) to the levels in the macromeres and mesomeres, which were virtually unchanged in comparison with the controls (Table 1).

**Table 1:** Effects of HpPKC inhibitors or activator on micromere-specific Ca^2+^ elevation (*P. lividus*)

Treatments	Micromeres	Macromeres	Mesomeres
Control	0.99 ± 0.02 (24)	0.91 ± 0.01 (23)	0.92 ± 0.02 (33)
5 μM HpPKC-I	0.91 ± 0.02 (17)	0.89 ± 0.02 (16)	0.90 ± 0.02 (26)
400 nM Gö6976	1.06 ± 0.02 (5)	0.98 ± 0.03 (5)	0.96 ± 0.02 (7)
8 nM PMA	0.76 ± 0.00 (2)	0.72 ± 0.04 (2)	0.69 ± 0.03 (2)

Ca^2+^ levels of each blastomere were reported as mean ± standard deviation (SD) of the ratios of fluorescence intensity of Ca^2+^-sensitive dye (calcium green BAPTA) to the intensity of the Ca^2+^-insensitive internal control dye (rhodamine red) in the same cell. The numbers of monitored cells were given in parentheses.

**Figure 6:**
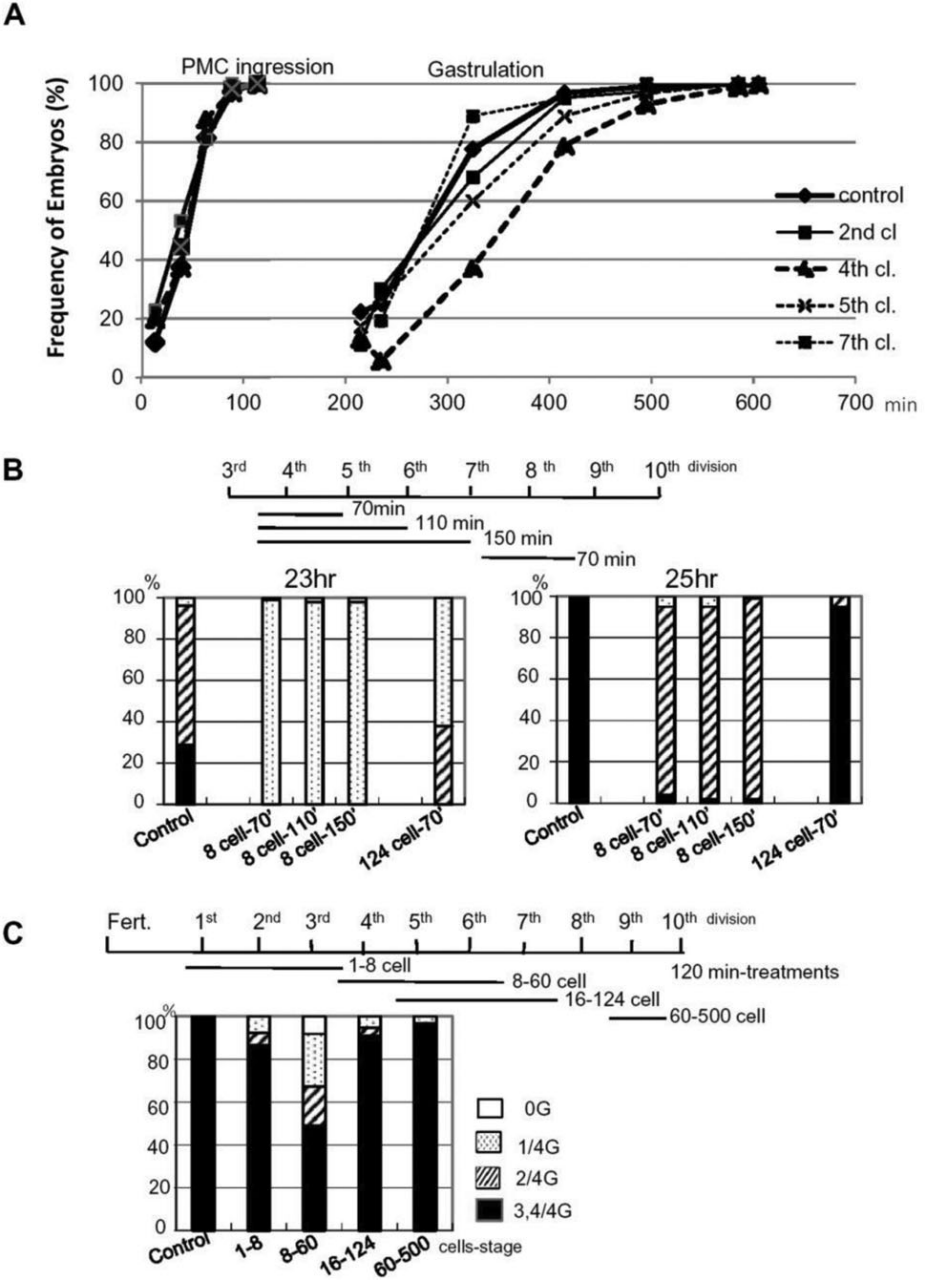
Inhibition of Ca^2+^infux and of PKC activity delayed gastrulation, but not PMC ingression. (*A*) *P. lividus* embryos were treated with 25 μM GdCl_3_ for 50 min starting from 15 min before the 2nd, 4th, 5th and 7th cleavages at room temperature. Cell cycles went on every 30–35 min. Control and GdCl_3_-treated embryos were both fixed at the same time, and the percentage of PMC-ingressed embryos or gastrulation-initiated embryos were calculated from circa 100 embryos from two or three independent experiments, respectively. (*B*, *C*) *H. pulcherrimus* embryos were treated with HpPKC-I at 6 μM (*B*) or 5 μM (*C*) during the period indicated by the horizontal bars. Gastrulation levels were estimated only from the side-viewed embryos. Embryos were cultured at 15°C (*B*), at 18°C (*C*). The cell cycles of these experiments were 45–48 min (*B*) and 39 min (*C*).

The earliest function of the micromeres appears to be endoderm induction, as the signal is emitted from the micromeres during the 16–60-cell stages (Ransick & Davidson, 1995). We have examined whether the Ca^2+^ influx and PKC activity are essential for this micromere signal. As the Ca^2+^ influx at the micromere formation is suppressed with GdCl_3_, an inhibitor of stretch-activated ion channels (Yazaki *et al*., 2004), we exposed *P. lividus* embryos to 25 μM GdCl_3_ and examined the frequency of the embryos that clearly exhibited the expected phenotypes: ingression of the primary mesenchyme cells (PMCs) and gastrulation (Fig. 6*A*). We found that gastrulation was delayed by GdCl_3_ when it was applied at the 4th and 5th cleavages, but not before or after the period. When GdCl_3_ was added from the 5th cleavage, which included micromere cleavage to form small and large micromeres during treatment, gastrulation was delayed to a lesser extent than when treatment was carried out at the 4th cleavage (Fig. 6*A*). By contrast, the ingression of PMCs into the blastocoel was not altered in all treatment schemes.

Similar to finding with GdCl_3_, HpPKC-I affected the timing of gastrulation as shown with *H. pulcherrimus* embryos exposed to 6 μM HpPKC-I (Fig. 6*B*, *C*). To quantify the progression of gastrulation, we rated the embryos into four grades based on the length of the invaginated archenteron: 0G, uninvaginated embryo; 1/4G, embryo with an indent at the vegetal pole; 2/4G, embryo with the archenteron elongated to the midpoint; 4/4G, embryo with the archenteron attained to the animal pole. The control embryos underwent gastrulation by 23 h (Fig. 6*B*). About 30% of embryos nearly completed gastrulation (3/4G + 4/4G), and the rest elongated their archenteron and arrived at the 2/4G stage. In contrast, the embryos exposed to HpPKC-I barely started gastrulation by 23 h. Two hours later (Fig. 6*B*, at 25 h), all the control embryos had finished gastrulation, but the embryos treated with HpPKC-I were still gastrulating (most embryos were estimated to be at 2/4G). The extent of the delay was not changed by the duration of the treatment, from 70 min (8–16-cell stage) to 150 min (8–60-cell stage). Thus, the delay of gastrulation by HpPKC-I appeared to have a critical period of about 70 min that was between the 8-cell and 16-cell stages. Indeed, inhibition of PKC from the 7th cleavage (124-cell stage) caused no delay in gastrulation (Fig. 6*B*), whereas the largest delay in gastrulation was provoked by the treatment during the 8–60-cell stages (Fig. 6*C*). In contrast, in all these conditions, PMC ingression took place on a normal schedule (data not shown), analogous to the results obtained with GdCl_3_ (Fig. 6*A*). Hence, inhibition of the stretch-activated Ca^2+^ channel (GdCl_3_) and PKC (HpPKC-I) both led to a delay in gastrulation but not to alteration of the micromere specifications.

### HpPKC inhibitor enlarged the oral ectoderm in both *H. pulcherrimus* and *P. lividus* embryos without affecting mesoendoderm specification

**Figure 7:**
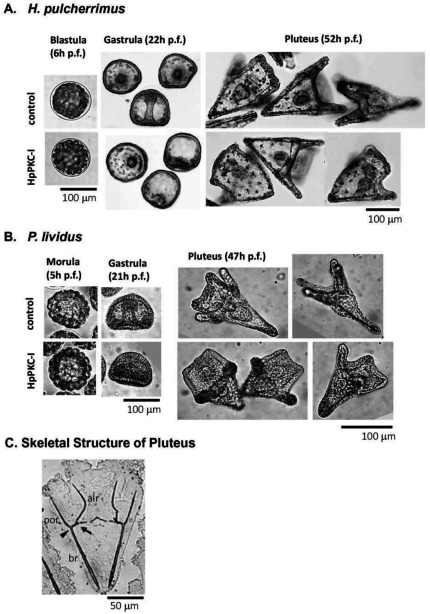
HpPKC inhibitor caused similar morphological changes on embryos of *H. pulcherrimus* and *P. lividus*. Embryos were treated with 5 μM HpPKC-I from 20 min before the 4th cleavage to 20 min after the 6th cleavage. This period was about 120 min for *H. pulcherrimus* culturing at 18°C and 100 min for *P. lividus* at 23°C. Control and HpPKC-I-treated embryos. (*A*) *H. pulcherrimus* embryos; early blastulae at 6 h post fertilization (p.f.), gastrulae at 22 h, and plutei at 52 h (*B*) *P. lividus*; morulae at 5 h, gastrulae at 21 h, and plutei at 47 h. (*C*) Names of the skeletal parts of the pluteus.

We have studied how HpPKC-I would affect the development of the embryo at later stages. To this end, embryos of *H. pulcherrimus* and *P. lividus* were treated with 5 μM HpPKC-I (Fig. 7). In comparison with the control, the embryos treated with HpPKC-I displayed: (i) disruptions of the cell-cell contact in the early blastula; (ii) delay of gastrulation; and (iii) enlargement of the oral ectoderm in the pluteus. However, the gut developed normal in these embryos, and the pigment cells were well discernible. When quartet micromeres isolated from a 16-cell stage embryo were recombined to an animal cap, the plutei that derived from the recombinant embryo displayed a normal distribution of alkaline phosphatase-positive cells (gut marker) and formation of functional guts regardless of pretreatment with HpPKC-I (data not shown). Thus, HpPKC-I does not appear to interfere with the ability of micromeres to induce mesoderm. However, HpPKC-I caused morphological shifts at the pluteus stage. When the skeletal size of plutei was measured with regard to the post-oral rod (por), body rod (br) and antero-lateral rod (alr) (nomenclature identified in Fig. 7*C*), the total length of the rod structures in the embryos treated with HpPKC-I was about 10% reduced compared with the control embryos, while the skeleton supporting oral ectoderm, alr, remained the same (Table 2). Thus, the effects of HpPKC-I on the skeleton size were restricted to br and por in the aboral ectoderm region. In contrast, LiCl showed a tendency to suppress skeletogenesis as a whole, whereas another PKC inhibitor, Gö6976, enhanced the growth of skeletons at the concentration used. Instead, the addition of PKC activator, PMA made no difference.

**Table 2:** Effects of PKC modulators on the formation of the skeletal rods in the pluteus (*P. lividus*)

	Body rods (Br)	Post-oral rods (Por)	Antero-lateral rods (Alr)	Total length (μm)
Control	103.5 ± 0.95 (102)	51.1 ± 0.90 (115)	64.0 ± 0.67 (118)	219
HpPKC-I	91.5 ± 0.67 (132)	42.2 ± 0.90 (118)	63.8 ± 0.67 (128)	198
Gö6976	103.2 ± 0.53 (163)	61.2 ± 0.59 (145)	75.9 ± 0.73 (146)	240
PMA	103.5 ± 0.39 (129)	55.9 ± 0.76 (125)	68.8 ± 0.56 (130)	228
LiCl	101.2 ± 0.71 (85)	45.4 ± 0.72 (77)	56.5 ± 0.99 (72)	203

In total, 5 μM HpPKC-I and 400 nM Gö6976 were added to embryos 20 min before the 4th cleavage; 8 nM PMA was added to the 16-cell stage embryos for 20 min, and 40 mM LiCl to the embryos at the 4-cell to 60-cell stages (2.5 h). The numbers of monitored cells are given in parentheses.

**Figure 8:**
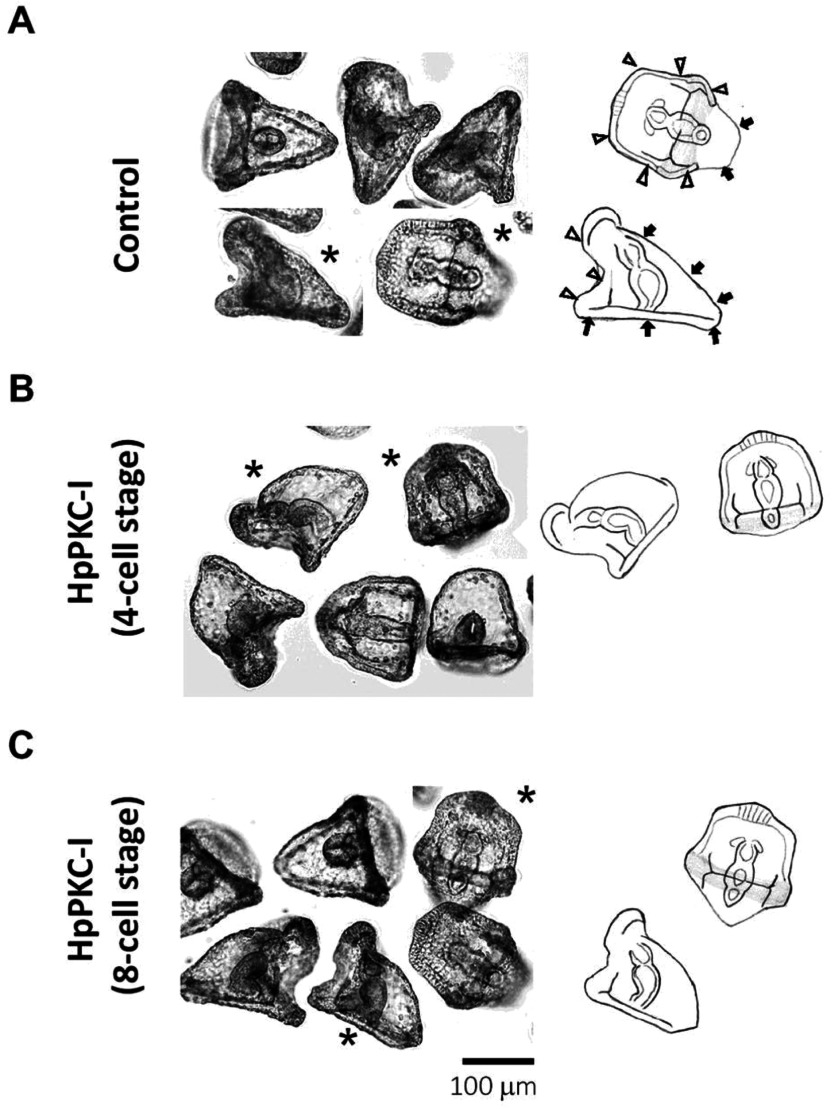
Morphological changes of *H. pulcherrimus* embryos treated with HpPKC inhibitor before the 8-cell stage. (*A*) Control embryos. (*B*) Embryos treated with 5 μM HpPKC-I starting from the 4-cell stage (20 min before the 3rd cleavage) to the end of 8-cell stage. (*C*) Embryos treated with 5 μM HpPKC starting from 20 min before the 4th cleavage to the end of 16-cell stage. Embryos were cultured at 18°C. The hand-drawn delineations on the right panels represent the embryos marked with asterisks in (*A–C*). Oral ectoderm area and aboral area were indicated by white arrowheads and black arrows, respectively.

As mentioned for Fig. 6*C*, when HpPKC-I was added to the embryos by the 8-cell stage, the timing of gastrulation was not changed, but it was noted that the shape of embryos seemed to be changed considerably (Fig. 8*B*). To discriminate the shape clearly, the embryos marked with asterisks in the left panels were delineated on the right to indicate the oral (arrowheads) and the aboral ectoderms (arrow) in the normal embryos (Fig. 8). When HpPKC-I was added before the 3rd cleavage (4-cell stage), the embryo displayed a much decreased aboral ectoderm in comparison with the control, forming embryos in which the oral ectoderm was disproportionally larger (Fig. 8*B*). Similar morphological changes were observed in the embryos treated with HpPKC-I after the 4th cleavage but to a lesser extent (Fig. 8*C*). Again, added after the 7th cleavage, HpPKC-I had no effect on the morphology of the embryos (data not shown).

### qPCR analysis of the embryos treated with the HpPKC inhibitor

To study the effect of PKC on the expression of the pivotal genes involved in early embryonic development, we examined the changes in levels of the 17 transcripts with or without treatment with HpPKC-I. As summarized in Table 3, the genes normally expressed in the oral ectoderm, *Nodal, Deadringer* and *Lim*, markedly increased their expression after the embryos were exposed to HpPKC-I, while the transcripts of the aboral ectoderm genes, *NK2.2* and *Coquilette*, decreased. In accordance with the morphology data (Table 2), the skeletogenic genes also decreased in expression. However, we need to pay special attention to those genes whose expression is linked to the timing of gastrulation. As the addition of HpPKC-I delayed gastrulation, the genes linked to gastrulation are likely to display an altered expression profile in response to HpPKC-I. As expected, the level of *Brain1/2/4*, a transcription factor that is expressed at the initiation of gastrulation and controls *endo16* (Yuh *et al*., 2005), was much lower after HpPKC-I treatment (Table 3). We have also found that HpPKC-I increased *Nodal* but suppressed *Deadringer* (*Dri*) transcripts. Together with *Lim*, *Nodal* and *Deadringer* (*Dri*) are involved in the development of oral ectoderm, but the expression times of these genes are known to be slightly different. In the beginning of gastrulation, *Nodal* declines by half (Duboc *et al*., 2004), but *Dri* increases (Amore *et al*., 2003). Likewise, genes involved in aboral ectoderm formation (*NK2.2* and *Coquilette*) are expressed from the blastula stage (Takacs *et al*., 2004; Croce *et al*., 2003), and accordingly we have found that HpPKC-I treatment suppressed their expression. Thus, our data with HpPKC-I are in line with these previous observations, and suggest that PKC signalling may contribute to the fine regulation of gene expression before gastrulation.

**Table 3:** qPCR analysis of embryos treated with HpPKC inhibitor

	Gastrula	Pluteus	
			
Genes	20 h	23 h	43 h
Endomesodermal transcription factors and signal components			
*Delta*	NS	NS	NS
*Gcm*	−2.73	NS	NS
*Fox A*	NS	NS	NS
*Bra*	NS	NS	NS
*Blimp/Krox*	NS	NS	+1.84
*Brain1/2/4*	−4.78	−3.91	NS
PMC ingression and skeletogenic genes			
*G*-*cadherin*	NS	−1.81	NS
*Sm50*	−2.71	−5.26	NS
*Sm37*	−2.40	NS	NS
*Sm30*	−2.34	−1.92	NS
Regulator genes of O–A axis formation			
*Nodal*	+3.81	NS	NS
*Deadringer* (*Dri*)	−5.44	−4.00	+3.32
*Lim*	NS	NS	+3.22
*Gsc*	−2.02	−2.46	NS
*FoxG*	−1.95	−1.89	NS
*NK2.2*	−4.40	−1.70	NS
*Coquilette*	−2.53	−1.79	NS

Embryos were treated with 5 μM HpPKC-I starting from 20 min before the 4th cleavage until 20 min after the 6th cleavage. Embryos were harvested at time points 20 h (early gastrula), 23 h (late gastrula or prism) and 43 h (two-armed pluteus) post fertilization. The D values defined in Methods and materials were calculated from the results of qPCR and reported in positive and negative numbers that respectively represent increases and decreases of the transcripts after the treatment with the inhibitor. NS, not significant (*P* > 0.05).

## Discussion

In the present study, we have demonstrated that the PKC pathway plays an important role during the early embryonic development of sea urchin. Suggestive of a conserved mechanism of control, the PKCs from the two species of sea urchins (*H. pulcherrimus* and *P. lividus*) shared the same myristoylated pseudosubstrate region that we have employed as a specific PKC inhibitor (HpPKC-I). HpPKC-I was highly effective and completely blocked cell division at 7 μM concentration. At 5 μM, phalloidin-stained F-actin filaments were apparently released from the plasma membrane region, disrupting the intercellular adhesion system (Figs. 1 and 7). Oral ectoderm specifications were also affected by HpPKC-I in both embryos (Fig. 7). Our studies using HpPKC-I have thus revealed that the PKC pathway is involved in the regulation of many aspects of early development such as cytokinesis, dynamic rearrangement of the actin cytoskeleton, nucleation of β-catenin, gastrulation, intracellular Ca^2+^ signalling, and the expression of the genes accountable for the formation of oral ectoderm. Taken together with previous findings that blastomeres of 16-cell stage sea urchin embryos have Ca^2+^ influx (Yazaki, 2001), the results of our studies using HpPKC-I suggested the Ca^2+^/DAG-dependent protein kinase PKC as the central piece of the signalling pathway that links the intracellular Ca^2+^ increase to various downstream events in early embryonic development.

Sea urchin embryos specify cell fates along two embryonic axes; animal–vegetal (AV) axis and oral–aboral (OA) axis. Both axes are initially inherent in the cytoarchitecture of the unfertilized egg (Boveri, 1901; Hörstadius, 1939; Coffman & Davidson, 2001; Croce *et al*., 2011). β-Catenin is a maternally expressed protein, and its nuclear entrance in the blastomeres sets the tone for the AV axis for cell specification (Wikramanayake *et al*., 1998; Logan *et al*., 1999; Croce *et al*., 2011). Nuclear entrance of β-catenin takes place in the micromeres and the macromeres (Fig. 4) on the vegetal side, but not on the animal side (Miller & McClay, 1997a; Logan *et al*, 1999). One of our major findings in this study is that this tight cell-specific control of nuclear localization of β-catenin is deregulated by the inhibition of PKC, as 5 μM HpPKC-I induced nuclear entrance of β-catenin in all cells at the 16-cell stage (Fig. 5*D*, *E*). In addition, we have found that the initial nuclear localization of β-catenin takes place in the macromeres (Figs. 4*A* and 5*A*). While the significance of this finding is not entirely clear, our result is at variance with a previous study using another sea urchin species *L. variegatus* in which the initial nuclear entry of β-catenin was reported to take place in the micromeres at the 16-cell stage (Logan *et al*., 1999). The specificity of our antibody against β-catenin of *H. pulcherrimus* was demonstrated in western blot analysis, which showed a single band of 103 kDa (Fig. 2*B*) that was in general agreement with the molecular weights of the β-catenins in *Xenopus* (92 kDa) (McCrea & Gumbiner, 1991) and in starfish embryos (100 kDa) (Miyawaki *et al*., 2003). Our antibody also visualized the characteristic cytoplasmic and membrane distributions of β-catenin reported in *L. variegatus* (Miller & McClay, 1997a) up to the 4th cleavage. Thus, this difference does not seem to represent a technical issue on our part. We have checked the subcellular localization of β-catenin in the context of mitotic cell cycle (Fig. 3), and confirmed that nuclear entrance of β-catenin first occurred in the macromeres at the 16-cell stage and then expanded to micromeres at the 32-cell stage, whereas nuclear localization of β-catenin in the micromeres clearly occurred only after the 5th cleavage (Fig. 4). Hence, whether this subtle difference in *H. pulcherrimus* and *L. variegatus* arises from a real biological difference in the two sea urchin species or simply reflects methodological limitations is yet to be resolved.

The results of our experiment using HpPKC-I raised intriguing questions as to how PKC regulates subcellular distribution of β-catenin, and as to the roles of nuclear β-catenin. In sea urchin embryos, it has been proposed that the specific nuclear entrance of β-catenin in the cells on the vegetal side is mediated by glycogen synthase kinase 3β (GSK-3β), which is a serine/threonine kinase comprising the Wnt pathway. In line with this idea, Lhomond *et al*., (2012) reported that Wnt6 and its receptor Frizzled 1/2/7 selectively expressed in macromeres were responsible for nuclear localization of β-catenin in macromere daughter cells after the 5th cleavage. In view of the fact that the Wnt/Ca^2+^ signalling pathway activates Ca^2+^-dependent kinases such as PKC and CAMK II in vertebrates (Kühl *et al*., 2000), it is conceivable that PKC may serve as a downstream effector of Wnt and preclude the nuclear entrance of β-catenin in blastomeres on the animal side in sea urchin embryos. Indeed, it has been reported that PKC inactivates GSK-3β by phosphorylation in test tubes and in fibroblasts (Goode *et al*., 1992; Cook *et al*., 1996). In addition, β-catenin is phosphorylated by the maternally expressed GSK-3β and rapidly degenerated in blastomeres on the animal side of the 16-cell stage sea urchin embryo, whereas β-catenin is stable in the vegetal cells because GSK-3β is inactivated by Dishevelled (Dsh) protein, a transducer of the canonical Wnt pathway (Weitzel *et al*., 2004; Croce *et al*., 2011). The idea that the subcellular localization of β-catenin may be mediated by GSK-3β was further supported by the results of our experiment using LiCl, which has been reported to inhibit GSK-3β activity *in vitro* (Klein & Melton, 1996) and is thus expected to exert the same effect that PKC has on GSK-3β. We found that the PKC activator PMA and LiCl both stabilized β-catenin in the cytoplasm, whereas 5 μM HpPKC-I appreciably reduced β-catenin in the cytoplasm (Fig. 5*D*, *E*).

In view of the fact that β-catenin entering the nucleus functions as a transcriptional activator of target genes (Miller *et al*., 1999), it is tempting to speculate that β-catenin in the nucleus of blastomeres on the vegetal side might lead to production of inductive factors that determine the vegetal cell fates (McClay *et al*., 2000). A paired homeodomain transcription factor pmar-1 is expressed exclusively in micromeres and is known to be a direct target of the β-catenin/TCF complex (Oliveri *et al*., 2002). Pmar1 in turn accelerates micromere-selective degradation of SoxB1, which is an ‘animalizing’ transcription factor counteracting β-catenin and thereby carving the patterning of cell fates along the AV axis (Oliveri *et al*., 2003, Angerer *et al*., 2005). Thus, deregulation of β-catenin expression or its translocation to the nucleus was expected to result in alteration of the pattern formation along the AV axis. Indeed, when β-catenin was sequestered in the plasma membrane by overexpression of the intracellular domain of cadherin (ΔLvG-cadherin), (Logan *et al*., 1999), the failure of β-catenin to enter the nucleus led to development of animalized embryos without formation of endoderm and mesoderm. Overexpression of ΔLvG-cadherin also inhibited SpWnt8 expression in the micromeres, which is essential for endomesoderm formation (Wikramanayake *et al*., 2004). In the present study using HpPKC-I, we have found a converse situation in which β-catenin entered the nuclei of all blastomeres at the 16-cell stage (Fig. 5). Although HpPKC-I did not vegetalize the embryo, the treatment led to the development of embryos with reduced aboral ectoderm (Figs. 7 and 8), implying that the nuclear β-catenin in the cells on the animal side may have a functionally different meaning from that on the vegetal side.

Considering the results so far, our conceivable model would be that PKC activity contributes to the nuclear localization of β-catenin in the blastomeres of the vegetal side, establishing the AV axis. Indeed, PKC is thought to be present in the eggs throughout the developmental stages of sea urchin (Rakow & Shen, 1994); the early embryos exposed to 12-*O*-tetradecanoyl phorbol-13-acetate (TPA, also called PMA), an activator of PKC, promotes the development of endoderm and mesoderm (Livingston & Wilt, 1992). In our study, we have demonstrated that the same PKC activator increased β-catenin in the cytoplasm and enlarged the nuclear entrance of β-catenin more to the animal side, as was observed with LiCl (Fig. 5*B1*, *C1*). In support of the idea that GSK-3β-dependent modulation of β-catenin level is largely responsible for regional specification, overexpression of GSK-3β produced animalized embryos, while the kinase-dead GSK-3β markedly vegetalized embryos (Emily-Fenouil *et al*., 1998). Consistent with these findings, the major components of the Wnt pathway have also recently been discovered in sea urchin embryos, as SpWnt8 was shown to be selectively expressed in the micromeres at the 16-cell stage, and SpWnt5 in mesomeres and macromeres (Stamateris *et al*., 2010). However, what remains largely unresolved is how LiCl mediates vegetalization. We found that LiCl expanded the nuclear entrance of β-catenin to a more animal side (Fig 5*B*), leading to vegetalization of the sea urchin embryo (Logan *et al*., 1999). LiCl vegetalizes the prospective ectodermal cells, but the effect could be reversed by injection of myo-inositol (Livingston & Wilt, 1995). This might be because LiCl inhibits inositol monophosphatase activity (Berridge *et al*., 1989) and thereby reduces the InsP_3_ increase that normally takes place in the embryos during mesoderm induction (Maslansky *et al*., 1992; Ciapa & Maggio, 1993). Whether this InsP_3_-dependent pathway of the Li^+^ effect would converge with the aforementioned GSK-3β pathway is yet to be clarified.

Another important layer of control in early embryogenesis is the changes in intracellular Ca^2+^ levels in the blastomeres. We have reported previously a polarized distribution of L-type Ca^2+^ channels along the AV axis of the *P. lividus* blastula, and that those activities are linked to the cortical actin network (Yazaki *et al*., 1995). This channel was activated during M-phase of cell cycles, and displayed Ca^2+^ current in the mesomeres (7.4 ± 3.8 μA/cm^2^) and in the macromeres (2.3 ± 2.7 μA/cm^2^) at the 16-cell stage, but not in the micromeres (Dale *et al*., 1997). However, this ion channel might be irrelevant to the PKC pathway that we described. Whereas a L-type channel antagonist Nifedipine delayed cleavage and inhibited spicule formation, 5 μM HpPKC-I did not affect cell division, and the formation of spicule and gut was normal in these embryos (Fig. 6 and Table 2). Instead, the PKC pathway leading to the pattern determination appeared to involve the Ca^2+^ influx that takes place slightly later at the 4th cleavage through a stretch-dependent Ca^2+^ channels in the bulging-out region of the micromere and thereby elevates the intracellular Ca^2+^ levels in the micromeres (Yazaki, 2001; Yazaki *et al*., 2004). We found that the abolishment of micromere-specific Ca^2+^ elevation with HpPKC-I (Table 1) and the suppression of the micromere-specific Ca^2+^ influx with a blocker of stretch-activated ion channel, GdCl_3_, both led to delayed gastrulation, but not to a delay in ingression of micromere to the PMCs (Fig. 6). Thus, PKC inhibitor blocked the micromere signals without interfering with the self-differentiation of the micromeres. One speculation on the mechanism enabling this outcome is that PKC might contribute to the formation of the gap junction-like adherence junctions, and that the micromeres effect mesoendoderm specifications through these junctions with the macromeres during the 16–60-cell stages. Indeed, the inhibitor for gap junctions 1-octanol markedly delayed gastrulation when added at the 16-cell stage (*Temnopleurus hardwicki*) or even at the 16- to 60-cell stages (*H. pulcherrimus*) (Yazaki *et al*., 1999). Interestingly, after the 7th cleavage, by which the fully passable gap junctions were already established (Yazaki *et al*., 1999), PKC inhibition no longer affected gastrulation nor morphogenesis (Figs. 6 and 7).

In this context, it is noteworthy that HpPKC-I drastically changed the subplasmalemmal actin cytoskeleton (Fig. 1*C*). The observation that PKC activity may modulate actin dynamics in a subcellular-specific manner offers two intriguing implications. Firstly, the actin network subjacent the plasma membrane may modulate the functions of adherence junctions. Yazaki (1984, 1991) showed that the sea urchin blastomeres had two membrane domains: the egg-originated apical membrane being lined with cortical actin meshwork and the newly formed membrane by each cleavage that is virtually devoid of actin filaments. The desmosomes in the electron micrograph start in the cell junctions at the 4-cell stage (Spiegel & Howard, 1983), but they do not display a network of microfilaments emanating from the desmosomal plaques unlike in the adult epithelium. This unique feature of embryonic desmosomes might facilitate direct cell-to-cell communication. Secondly, the control of the subplasmalemmal actin meshwork by PKC might serve as a way to modulate the activity of the L-type channel and stretch-activated Ca^2+^ channels (Yazaki *et al*., 1995; Yazaki, 2001) in view of recent findings that the actin cytoskeleton may affect activities of the intracellular Ca^2+^ channels (reviewed in Chun *et al*., 2010).

In this study, we have also found that inhibition of PKC interfered with gene expression in later embryonic stages (Table 3). HpPKC-I decreased the expression of aboral ectoderm specification genes, *NK2.2* and *Coquilette*. The expression of Brain1/2/4, a regulator for midgut-specific transcriptions, was markedly delayed in agreement with the delay in gastrulation, but it was later expressed to form the gut with the same size and shape as in the control (Figs. 7 and 8). As for the skeletogenic genes, HpPKC-I repressed the skeletal matrix genes, Sm-30, Sm-37 and Sm-50. This finding exhibits a good correlation with the formation of a smaller skeleton (Table 2). Likewise, HpPKC-I suppressed *Deadringer* (*Dri*) gene (Table 3), which in sea urchin (*SpDri*) is first expressed in the precursor cells of PMCs but disappears altogether with PMCs ingression. Just before gastrulation, *SpDri* restarts its expression in the presumptive oral ectoderm (Amore *et al*., 2003). Thus, *SpDri* suppression in the gastrulae pretreated with HpPKC-I is in line with our observation of delayed gastrulation. Amore *et al*. (2003) found that expression of *SpDri* in the oral ectoderm is required to impart the pattering information to the skeletogenic PMCs that is needed for oral rod (alr) formation. A chimera that consisted of normal micromeres and micromere-deleted embryo using *SpDri* morpholino developed into an alr-deficient pluteus. In the present study, we have observed that, as a result of PKC inhibition during the 16–60-cell stages, alr was larger than the other skeletons, br and por (Table 2). Increases in *Dri* expression should guarantee this preferential growth of alr probably in the cells on the animal side (Table 2). We have also found that HpPKC-I induced a late increase in *Lim* transcripts by 43 h, in the early pluteus (Table 3). Overexpression of *HpLim1* directed all embryonic cells to differentiate into oral ectoderm (Kawasaki *et al*., 1999). *SpLim1* was expressed in the ciliated band along the oral and aboral ectoderm border with the endoderm of the pluteus (Su *et al*., 2009). We have also found that HpPKC-I increased expression of the oral ectoderm genes, *Nodal, Deadringer* (*Dri*) and *Lim*. Our result suggesting that *Nodal* gene is modulated by PKC activity is of keen interest, as *Nodal* is probably the earliest zygotic marker that determines OA polarity following the mitochondrial distribution gradient and the local redox state (Duboc *et al*., 2004; Coffman *et al*., 2004, 2009). To establish the OA axis, animal cells also need to receive the vegetal signals after the 4th division to express the aboral ectoderm (Kominami *et al*., 2006). Without the vegetal signals, the explant of the animal half develops into an epithelial ball consisting of neurogenic ectoderm, in which the OA axis fails to form due to the deficiency of aboral ectoderm expression, and the oral ectoderm marker molecules express all over the embryo (Hörstadius, 1975; Wikramanayake *et al*., 1995; Yaguchi *et al*., 2006). Interestingly, to express the aboral ectoderm in these embryos, proper expression of β-catenin and Otx are essential (Wikramanayake & Klein, 1997; Wikramanayake *et al*., 1998; Li *et al*., 1999), the latter of which is a transcription factor for aboral ectoderm genes (Mao *et al*., 1996). Thus, PKC seems to be implicated in the fine regulation of a variety of developmental genes at different times, and in part may well modulate cell fate and pattern formation in the other axis through *Nodal* and β-catenin. This situation might explain why the oral ectoderm specification was exaggerated if the embryo was treated with HpPKC-I before the 8-cell stage (Fig. 8), although the gastrulation timing and the gut morphology were not changed (Fig. 6*B*, *C*).

In summary, we propose a model in which Ca^2+^influx conducted and amplified through Wnt/Ca^2+^ (animal side) and Wnt/β-catenin signalling pathways (vegetal side) may add to activate PKC, whose enzyme activity is further enhanced by DAG that is produced by Ca^2+^-dependent phospholipase C. From the first cleavage, PKC in the sea urchin embryo may function in association with the actin cytoskeletons to direct various downstream events: (i) regulation of β-catenin localization; (ii) guiding intercellular communications by constructing cell adhesive structures by the 60-cell stage; and (iii) establishment of the embryonic axes, animal–vegetal and oral–aboral axes.

## References

[ref001] Amore G., Yavrouian R.G., Peterson K.J., Ransick A., McClay D.R. & Davidson E.H. (2003). *Spdeadringer*, a sea urchin embryo gene required separately in skeletogenic and oral ectoderm gene regulatory networks. Dev. Biol. 261, 55–81. 12941621 10.1016/s0012-1606(03)00278-1

[ref002] Angerer L.M., Newman L.A. & Angerer R.C. (2005). SoxB1 downregulation in vegetal lineages of sea urchin embryos is achieved by both transcriptional repression and selective protein turnover. Development 132, 999–1008. 15689377 10.1242/dev.01650

[ref003] Berridge M.J. (1993). Inositol trisphosphate and calcium signalling. Nature 361, 315–325. 8381210 10.1038/361315a0

[ref004] Berridge M.J., Downes C.P. & Hanley M.R. (1989). Neural and developmental actions of lithium: A unifying hypothesis. Cell 59, 411–9. 2553271 10.1016/0092-8674(89)90026-3

[ref005] Bienz M. (2005). β-Catenin: a pivot between cell adhesion and Wnt signalling. Curr Biol. 15, R64–7. 15668160 10.1016/j.cub.2004.12.058

[ref006] Boveri T. (1901). Uber die Polaritate des Seeigel-Eies. Verh. Phys.-Med. Ges. Wurzburg 34, 145–76.

[ref007] Chun J.T., Puppo A., Vasilev F., Gragnaniello G., Garante E. & Santella L. (2010). The biphasic increase of PIP2 in the fertilized eggs of starfish: new roles in actin polymerization and Ca^2+^ signaling. PLoS One 5, e14100. 21124897 10.1371/journal.pone.0014100 PMC2990714

[ref008] Ciapa B. & Maggio K. (1993). Effect of lithium on ionic balance and poly-phosphoinositide metabolism during larval vegetalization of the sea urchin *Paracentrotus lividus*. Dev. Biol. 159, 114–21. 8396053 10.1006/dbio.1993.1225

[ref009] Coffman J.A. & Davidson E.H. (2001). Oral–aboral axis specification in the sea urchin embryo I. Axis entrainment by respiratory asymmetry. Dev. Biol. 230, 18–28. 11161559 10.1006/dbio.2000.9996

[ref010] Coffman J.A., McCarthy J.J., Dickey-Sims C. & Robertson A.J. (2004). Oral–aboral axis specification in the sea urchin embryo. II. Mitochondrial distribution and redox state contribute to establishing polarity in *Strongylocentrotus purpuratus*. Dev. Biol. 273, 160–71. 15302605 10.1016/j.ydbio.2004.06.005

[ref011] Coffman J.A., Coluccio A., Planchart A. & Robertson A.J. (2009). Oral–aboral axis specification in the sea urchin embryo III. Role of mitochondrial redox signaling via H_2_O_2_. Dev. Biol. 330, 123–30. 19328778 10.1016/j.ydbio.2009.03.017 PMC2748885

[ref012] Cook D., Fry M.J., Hughes K., Sumathipala R., Woodgett J.R. & Dale T.C. (1996). Wingless inactivates glycogen synthase kinase-3 via an intracellular signaling pathway which involves a protein kinase C. EMBO J. 15, 4526–36. 8887544 PMC452182

[ref013] Croce J., Lhomond G. & Gache C. (2003). *Coquilette*, a sea urchin T-box gene of the Tbx2 subfamily, is expressed asymmetrically along the oral–aboral axis of the embryo and is involved in skeletogenesis. Mech. Dev. 120, 561–72. 12782273 10.1016/s0925-4773(03)00022-4

[ref014] Croce J., Range R., Wu S.Y., Miranda E., Lhomond G., Peng J.C., Lepage T. & McClay D.R. (2011). Wnt6 activates endoderm in the sea urchin gene regulatory network. Development 138, 3297–306. 21750039 10.1242/dev.058792 PMC3133919

[ref015] Dale B., Yazaki I. & Tosti E. (1997). Polarized distribution of L-type calcium channels in sea urchin embryos. Am. J. Physiol. 273, 822–5. 10.1152/ajpcell.1997.273.3.C822 9316401

[ref016] Davidson E.H., Rast J.P., Oliveri P., Ransick A., Calestani C., Yuh C-H., Minokawa T., Amore G., Hinman V., Arenas-Mena C., Otim O., Brown C.T., Livi C.B., Lee P.Y., Revilla R., Schilstra M.J., Clark P.J.C., Rust A.G., Pan Z., Arnone M.I., Rowen L., Cameron R.A., McClay D.R., Hood L. & Bolouri H. (2002). A provisional regulatory gene network for specification of endomesoderm in the sea urchin embryo. Dev. Biol. 246, 162–90. 12027441 10.1006/dbio.2002.0635

[ref017] Dickey-Sims C., Robertson A.J., Rupp D.E., McCarthy J.J. & Coffman J.A. (2005). Runx-dependent expression of PKC is critical for cell survival in the sea urchin embryo. BMC Biology 3, 18. 16076398 10.1186/1741-7007-3-18 PMC1187879

[ref018] Drummond A.H. & Raeburn C.A. (1984). The interaction of lithium with thyrotropin-releasing hormone-stimulated lipid metabolism in GH3 pituitary tumour cells. Enhancement of stimulated 1,2-diacylglycerol formation. Biochem J. 22, 29–36. 10.1042/bj2240129 PMC1144405 6439191

[ref019] Duboc V., Rottinger E., Besnardeau L. & Lepage T. (2004). *Nodal* and *BMP2/4* signaling organizes the oral–aboral axis of the sea urchin embryo. Dev. Cell 6, 397–410. 15030762 10.1016/s1534-5807(04)00056-5

[ref020] Eichholtz T., de Bont D.B.A., de Widt J., Liskamp R.M.J. & Ploegh H.L. (1993). A myristoylated pseudosubstrate peptide, a novel protein kinase C inhibitor. J. Biol. Chem. 268, 1982–6. 8420972

[ref021] Eliyahu E., Tsaadon A., Shtraizent N. & Shalgai R. (2005). The involvement of protein kinase C and actin filaments in cortical granule exocytosis in the rat. Reproduction 125, 161–70. 15695610 10.1530/rep.1.00424

[ref022] Emily-Fenouil F., Ghiglione C., Lhomond G., Lepage T. & Gache C. (1998). GSK3/shaggy mediates patterning along the animal–vegetal axis of the sea urchin embryo. Development 125, 2489–98. 9609832 10.1242/dev.125.13.2489

[ref023] Fuchikami T., Mitsunaga-Nakatsubo K., Amemiya S., Hosomi T., Watanabe T., Kurokawa D., Kataoka M., Harada Y., Satoh N., Kusunoki S., Takata K., Shimotori T., Yamamoto T., Sakamoto N., Shimada H. & Akasaka K. (2002). *T*-*brain* homologue (*HpTb*) is involved in the archenteron induction signals of micromere descendant cells in the sea urchin embryo. Development 129, 5205–16. 12399312 10.1242/dev.129.22.5205

[ref024] Goode N., Hughes K., Woodgett J.R. & Parker P.J. (1992). Differential regulation of glycogen synthase kinase-3β by protein kinase C isotypes. J. Biol. Chem. 267, 16878–82. 1324914

[ref025] Gumbiner B.M. (1996). The molecular basis of tissue architecture and morphogenesis. Cell 84, 345–57. 8608588 10.1016/s0092-8674(00)81279-9

[ref026] Hörstadius S. (1939). The mechanism of sea urchin development, studied by operative methods. Biol. Rev. Camb. Phil. Soc. 14, 132–79.

[ref027] Hörstadius S. (1975). Isolation and transplantation experiments In The Sea Urchin Embryo Biochemistry and Morphogenesis (ed. G. Czihak), pp. 364–406 Springer-Verlag Berlin. Heidelberg. New York.

[ref028] Kawasaki T., Mitsunaga-Nakatsubo K., Takeda K., Akasaka K. & Shimada H. (1999). *Lim 1* related homeobox gene (*HpLim 1*) expressed in sea urchin embryos. Dev. Growth Differ. 41, 273–282. 10400389 10.1046/j.1440-169x.1999.413432.x

[ref029] Klein P.S. & Melton D.A. (1996). A molecular mechanism for the effect of lithium on development. Proc. Natl. Acad. Sci. USA 93, 8455–9. 8710892 10.1073/pnas.93.16.8455 PMC38692

[ref030] Kominami T., Akagawa M. & Takata H. (2006). Subequatorial cytoplasm plays an important role in ectoderm patterning in the sea urchin embryo. Dev. Growth Differ. 48, 101–15. 16512854 10.1111/j.1440-169X.2006.00850.x

[ref031] Kühl M., Sheldahl L.C., Park M., Miller J.R. & Moon R.T. (2000). The Wnt/Ca^2+^ signaling pathway takes shape. Trends Genet. 16, 279–82. 10858654 10.1016/s0168-9525(00)02028-x

[ref032] Kyozuka K., Chun J.T., Puppo A., Gragnaniello G., Garante E. & Santella L. (2008). Actin cytoskeleton modulates calcium signaling during maturation of starfish oocytes. Dev. Biol. 320, 426–35. 18599031 10.1016/j.ydbio.2008.05.549

[ref033] Lhomond G., McClay D.R., Gache C. & Croce J.C. (2012). Frizzled1/2/7 signaling directs β-catenin nuclearisation and initiates endoderm specification in macromeres during sea urchin embryogenesis. Development 139, 816–25. 22274701 10.1242/dev.072215 PMC3265065

[ref034] Li X., Wikramanayake A.H. & Klein W.H. (1999). Requirement of SpOtx in cell fate decisions in the sea urchin embryo and possible role as a mediator of β-catenin signaling. Dev. Biol. 212, 425–39. 10433832 10.1006/dbio.1999.9360

[ref035] Li X., Friedman A.B., Zhu W., Wang L., Boswell S., May R.S., Davis L.L. & Jope R.S. (2007) Lithium regulates glycogen synthase kinase-3β in human peripheral blood mononuclear cells: implication in the treatment of bipolar disorder. Biol. Psychiatry 61, 216–22. 16806104 10.1016/j.biopsych.2006.02.027 PMC1853347

[ref036] Livingston B.T. & Wilt F.H. (1992). Phorbol esters alter cell fate during development of sea urchin embryos. J. Cell Biol. 119, 1641–8. 1469053 10.1083/jcb.119.6.1641 PMC2289755

[ref037] Livingston B.T. & Wilt F.H. (1995). Injection of myo-inositol reverses the effects of lithium on sea urchin blastomeres. Develop. Growth Differ. 37, 539–43. 10.1046/j.1440-169X.1995.t01-4-00008.x 37280965

[ref038] Logan C.Y., Miller J.R., Ferkowicz M.J. & McClay D.R. (1999). Nuclear β-catenin required to specify vegetal cell fates in the sea urchin embryo. Development 126, 345–57. 9847248 10.1242/dev.126.2.345

[ref039] MacDonald B.T., Tamai K. & He X. (2009). Wnt/beta-catenin signaling: components, mechanisms, and diseases. Dev. Cell 17, 9–26. 19619488 10.1016/j.devcel.2009.06.016 PMC2861485

[ref040] Mao C-A., Wikramanayake A.H., Gan L., Chuang C-K., Summers R.G. & Klein W.H. (1996). Altering cell fates in sea urchin embryos by overexpressing SpOtx, an orthodenticle-related protein. Development 122, 1489–98. 8625836 10.1242/dev.122.5.1489

[ref041] Maslansky J.A., Leshko L. & Busa W.B. (1992). Lithium-sensitive production of inositol phosphate during amphibian embryonic mesoderm induction. Science 256, 243–55. 1314424 10.1126/science.1314424

[ref042] McClay D.R., Peterson R.E., Range R.C., Winter-Vann A.M. & Ferkowicz M.J. (2000). A micromere induction signal is activated by β-catenin and acts through notch to initiate specification of secondary mesenchyme cells in the sea urchin embryo. Development 127, 5113–22. 11060237 10.1242/dev.127.23.5113

[ref043] McCrea P.D. & Gumbiner B. (1991) Purification of a 92-kDa cytoplasmic protein tightly associated with the cell–cell adhesion molecule E-cadherin (uvomorulin): characterization and extractability of the protein complex from the cell cytostructure. J. Biol. Chem. 266, 4514–20. 1999432

[ref044] Miller J.R. & McClay D.R. (1997a). Changes in the pattern of adherens junction-associated β-catenin accompany morphogenesis in the sea urchin embryo. Dev. Biol. 192, 310–22. 9441670 10.1006/dbio.1997.8739

[ref045] Miller J.R. & McClay D.R. (1997b). Characterization of the role of cadherin in regulating cell adhesion during sea urchin development. Dev. Biol. 192, 323–39. 9441671 10.1006/dbio.1997.8740

[ref046] Miller J.R., Hocking A.M., Brown J.D. & Moon R.T. (1999). Mechanism and function of signal transduction by the Wnt/beta-catenin and Wnt/Ca^2+^ pathways. Oncogene 18, 7860–72. 10630639 10.1038/sj.onc.1203245

[ref047] Mitsunaga K., Shinohara S. & Yasumasu I. (1990). Probable contribution of protein phosphorylation by protein kinase C to spicule formation in sea urchin embryos. Develop. Growth Differ. 32, 335–42. 10.1111/j.1440-169X.1990.00335.x 37282312

[ref048] Miyawaki K., Yamamoto M., Saito K., Saito S., Kobayashi N. & Matsuda S. (2003). Nuclear localization of β-catenin in vegetal pole cells during early embryogenesis of the starfish *Asterina pectinifera*. Dev. Growth Differ. 45, 121–8. 12752500 10.1034/j.1600-0854.2004.00681.x

[ref049] Nishizuka Y. (1984). Turnover of inositol phospholipids and signal transduction. Science 225, 1365–70. 6147898 10.1126/science.6147898

[ref050] Okazaki K. (1975). Normal development to metamorphosis In The Sea Urchin Embryo Biochemistry and Morphogenesis (ed. G. Czihak), pp. 177–216 Springer-Verlag Berlin Heidelberg New York.

[ref051] Oliveri P., Carrick D.M. & Davidson E.H. (2002). A regulatory gene network that directs micromere specification in the sea urchin embryo. Dev. Biol. 246, 209–28. 12027443 10.1006/dbio.2002.0627

[ref052] Oliveri P., Davidson E.H. & McClay D.R. (2003). Activation of *Pmar1* controls specification of micromeres in the sea urchin embryo. Dev. Biol. 258, 32–43. 12781680 10.1016/s0012-1606(03)00108-8

[ref053] Rakow T.L. and Shen S.S. (1994). Molecular cloning and characterization of protein kinase C from the sea urchin *Lytechinus pictus*. Dev. Growth Differ. 36, 489–97. 10.1111/j.1440-169X.1994.00489.x 37281064

[ref054] Ransick A. & Davidson E.H. (1993). A complete second gut induced by transplanted micromeres in the sea urchin embryo. Science 259, 1134–8. 8438164 10.1126/science.8438164

[ref055] Ransick A. and Davidson E.H. (1995). Micromeres are required for normal vegetal plate specification in sea urchin embryos. Development 121, 3215–22. 7588056 10.1242/dev.121.10.3215

[ref056] Shen S.H. & Buck W.R. (1990). A synthetic peptide of pseudosubstrate domain of protein kinase C blocks cytoplasmic alkalization during activation of the sea urchin egg. Dev. Biol. 140, 272–80. 2373253 10.1016/0012-1606(90)90077-v

[ref057] Spiegel E. & Howard L. (1983). Development of cell junctions in sea urchin embryos. J. Cell Sci. 62, 27–48. 6684666 10.1242/jcs.62.1.27

[ref058] Stamateris R.E., Rafiq K. & Ettensohn C.A. (2010). The expression and distribution of Wnt and Wnt receptor mRNAs during early sea urchin development. Gene Expr. Patterns 10, 60–4. 19853669 10.1016/j.gep.2009.10.004

[ref059] Su Y-H., Li E., Geiss G.K., Longabaugh W.J.R., Kramer A. & Davidson E.H. (2009). A perturbation model of the gene regulatory network for oral and aboral ectoderm specification in the sea urchin embryo. Dev. Biol. 329, 410–21. 19268450 10.1016/j.ydbio.2009.02.029 PMC2677136

[ref060] Takacs C.M., Amore G., Oliveri P., Pouska A.J., Wang D., Burke R.D. & Peterson K.J. (2004). Expression of an NK2 homeodomain gene in the apical ectoderm defines a new territory in the early sea urchin embryo. Dev. Biol. 269, 152–64. 15081364 10.1016/j.ydbio.2004.01.023

[ref061] Takeichi M. (1991). Cadherin cell adhesion receptors as a morphogenetic regulator. Science 251, 1451–5. 2006419 10.1126/science.2006419

[ref062] Weitzel H.E., Illiew M.R., Byrum C.A., Xu R., Wikramanayake A.H. & Ettensohn C.A. (2004). Differential stability of β-catenin along the animal–vegetal axis of the sea urchin embryo mediated by disheveled. Development 131, 2947–56. 15151983 10.1242/dev.01152

[ref063] Wikramanayake A.H. & Klein W.H. (1997). Multiple signaling events specify ectoderm and pattern the oral–aboral axis in the sea urchin embryo. Development 124, 13–20. 9006063 10.1242/dev.124.1.13

[ref064] Wikramanayake A.H., Brandhorst B.P. & Klein W.H. (1995). Autonomous and non-autonomous differentiation of ectoderm in different sea urchin species. Development 121,1497–505. 7789279 10.1242/dev.121.5.1497

[ref065] Wikramanayake A.H., Huang L. and Klein W.H. (1998). β-catenin is essential for patterning the maternally specified animal–vegetal axis in the sea urchin embryo. Proc. Natl. Acad. Sci. USA 95, 9343–8. 9689082 10.1073/pnas.95.16.9343 PMC21340

[ref066] Wikramanayake A.H., Peterson R., Chen J., Huang L., Bince J.M., McClay R.D., & Klein W.H. (2004). Nuclear β-catenin-dependent Wnt8 signaling in vegetal cells of the early sea urchin embryo regulates gastrulation and differentiation of endoderm and mesodermal cell lineages. Genesis 39, 194–205. 15282746 10.1002/gene.20045

[ref067] Wilson E.B. (1937). The Cell in Development and Heredity. New York: The Macmillan Company.

[ref068] Yaguchi S., Yaguchi J. & Burke R.D. (2006). Specification of ectoderm restricts the size of the animal plate and patterns neurogenesis in sea urchin embryos. Development 133, 2337–46. 16687447 10.1242/dev.02396

[ref069] Yang X.C. & Sacks F. (1989). Block of stretch-activated ion channels in *Xenopus* oocytes by gadolinium and calcium ions. Science 243, 1068–71. 2466333 10.1126/science.2466333

[ref070] Yazaki I. (1984). The egg originated and local distribution of the surface of sea-urchin embryo cells detected by immunofluorescence. Acta Embryol. Morphol. Exper. 5, 3–22.

[ref071] Yazaki I. (1991). Polarization of the surface membrane and cortical layer of sea urchin blastomeres, and its inhibition by cytochalasin B. Dev. Growth Differ. 33, 267–76. 10.1111/j.1440-169X.1991.00267.x 37280809

[ref072] Yazaki I. (2001). Ca^2+^ in specification of vegetal cell fate in early sea urchin embryos. J. Exp. Biol. 204, 823–34. 11171406 10.1242/jeb.204.5.823

[ref073] Yazaki I., Tosti E. & Dale B. (1995). Cytoskeletal elements link calcium channel activity and the cell cycle in early sea urchin embryos. Development 121, 1827–31.

[ref074] Yazaki I., Dale B. & Tosti E. (1999). Functional gap junctions in the early sea urchin embryo are located to the vegetal pole. Dev. Biol. 212, 503–10. 10433838 10.1006/dbio.1999.9354

[ref075] Yazaki I., Abe M., Santella L. & Koyama Y. (2004). Mechanism of calcium elevation in the micromeres of sea urchin embryos. Biol. Cell 96, 153–67. 15050370 10.1016/j.biolcel.2003.11.009

[ref076] Yuh C-H., Dorman E.R. & Davidson E.H. (2005). *Brn1/2/4*, the predicted midgut regulator of the *endo 16* gene of the sea urchin embryo. Dev. Biol. 281, 286–98. 15893979 10.1016/j.ydbio.2005.02.034

